# Hospital Meetings

**Published:** 1904-07-16

**Authors:** 


### HOSPITAL MEETINGS.

### ST. GEORGE'S HOSPITAL.

#### QUARTERLY AND SPECIAL COURT.

AT the quarterly and special Court of Governors of St. George's Hospital, held on Friday, 8th inst., Dr. Theodore Duka was voted to the chair.

Minutes of the weekly board meetings during the past six months having been read and confirmed, new governors elected, and other routine business transacted, the Court became special, to receive the annual report for 1903, and the auditor's report, and the receipts and payments for the same period, and to make certain alterations in the laws.

In proposing the adoption of the report Mr. A. W. West, treasurer, commented on two points, *i.e.* the resignation of Mr. Timothy Holmes and the fact that, while the Hospital Sunday Fund had made adverse comments upon the expenditure of the hospital, their grant had been increased. Mr. Timothy Holmes, treasurer of the hospital since 1894, had resigned, and a warmly appreciative resolution had been passed thanking him for his eminent services, both to the hospital and to the medical school. Lord Windsor had been appointed in place of the late Duke of Richmond and Gordon, while the office of treasurer devolved upon Mr. A. William West, formerly deputy-treasurer.

The proposed alterations in the laws were then submitted by the treasurer, Mr. A. W. West and, with one exception, agreed to by the governors present. In Law 27, relating to the quorum at quarterly and special courts, the word "seven" was substituted for "nine," and the words "when the yearly accounts are to be disposed of, eleven governors shall be a quorum" were deleted. It was also agreed to delete from Law 37 the requisition that the physician or surgeon on the preceding week should make to the weekly board a report of the admission or non-admission of patients bringing in-patients' letters during that week. The proposal to give the resident medical officer a general supervision of the surgeries was negatived on grounds of impracticability.

The election of twenty-one governors, in addition to certain officers of the hospital, in accordance with Law 69, to form a committee to fill vacancies in any of the offices mentioned in that law which might occur during the ensuing twelve months, having taken place, the meeting closed with a vote of thanks to the Chair.

A general inspection of the hospital and in-patients took place on Tuesday, 5th inst., at noon.

